# (25*R*)-16β-Acet­oxy-3β-bromo-23′,26-ep­oxy-23′,25-dimethyl-5α-cholest-23,23′-en-6-one dichloro­methane monosolvate

**DOI:** 10.1107/S1600536812043590

**Published:** 2012-11-03

**Authors:** Susana Rincón, Rebeca Yépez, M. Eugenia Ochoa, Yliana López, Rosa Santillan, Norberto Farfán

**Affiliations:** a División de Posgrado, Instituto Tecnológico de Mérida, Avenida Tecnológico, Km 4.5 S/N C.P. 97118, Mérida Yucatán, Mexico; b Departamento de Química, CINVESTAV–IPN, Apdo. Postal 14-740, 07000 México, D.F., Mexico; c Instituto de Investigaciones Químico-Biológicas, Universidad Michoacana de San Nicolás de Hidalgo, Morelia, Michoacán, C.P. 58000, Mexico; d Facultad de Química, Departamento de Química Orgánica, Universidad Nacional Autónoma de México, México D.F., 04510, Mexico

## Abstract

The crystal structure of the title compound, C_31_H_45_BrO_5_·CH_2_Cl_2_, prepared in six steps from diosgenin, confirmed that the configurations of the stereogenic centers, positions 20*S* and 25*R*, remain unchanged during the reaction. The six-membered *A*, *B* and *C* rings have chair conformations. The five-membered ring *D* has an envelope conformation (with the methyl-substituted C atom fused to ring *C* as the flap) and the six-membered dihydro­pyran ring *E* adopts a twist-boat conformation. In the crystal, mol­ecules are linked *via* C—H⋯O and C—H⋯Cl hydrogen bonds, the latter involving the dichloro­methane solvent mol­ecule, forming a three-dimensional supra­molecular network.

## Related literature

For a review on saponins, see: Hostettmann & Marston (1995 ▶). For the use of spiro­stane sapogenins in the synthesis of biologically active compounds, see: Lee *et al.* (1976 ▶, 2009 ▶); Phillips & Shair (2007 ▶); Pettit *et al.* (1988 ▶). For compounds used in the sythesis and for various details of the synthetic procedure, see: Corey & Suggs (1975 ▶); Steele & Mosettig (1963 ▶); Iglesias-Arteaga *et al.* (1998 ▶); Monroe & Serota (1956 ▶); Rincón *et al.* (2006 ▶). For the crystal structure of a related steroidal compound containing bromine in the same position, see: Castro-Méndez *et al.* (2002 ▶). For standard bond lengths, see: Allen *et al.* (1987 ▶). For conformational analysis, see: Cremer & Pople (1975 ▶).
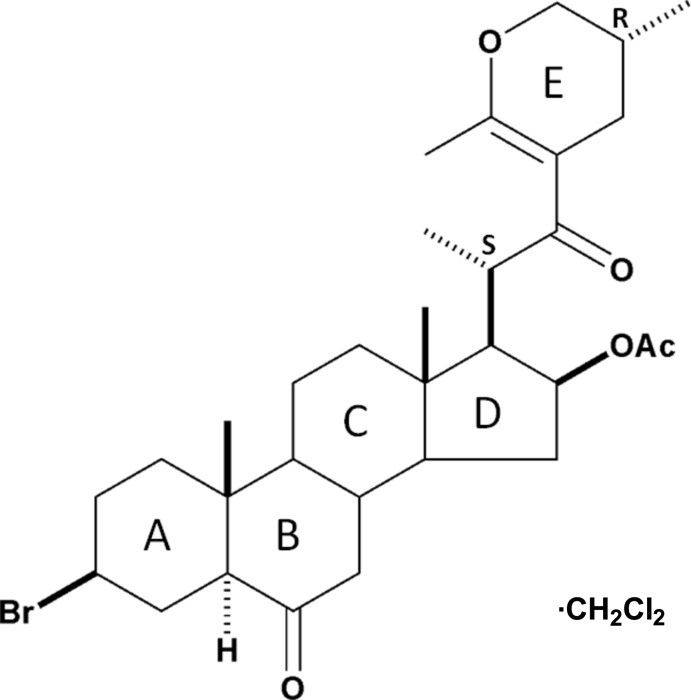


## Experimental

### 

#### Crystal data

C_31_H_45_BrO_5_·CH_2_Cl_2_
*M* *_r_* = 662.51
Orthorhombic, 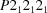

*a* = 7.4423 (1) Å
*b* = 15.6578 (2) Å
*c* = 26.8496 (3) Å
*V* = 3128.79 (7) Å^3^
*Z* = 4
Mo *K*α radiation
μ = 1.52 mm^−1^
*T* = 293 K
0.15 × 0.10 × 0.08 mm

#### Data collection

Nonius KappaCCD diffractometer
37310 measured reflections
7934 independent reflections
5668 reflections with *I* > 2σ(*I*)
*R*_int_ = 0.088

#### Refinement

*R*[*F* ^2^ > 2σ(*F* ^2^)] = 0.068
*wR*(*F* ^2^) = 0.181
*S* = 1.03
7934 reflections
362 parameters
H-atom parameters constrained
Δρ_max_ = 0.75 e Å^−3^
Δρ_min_ = −0.68 e Å^−3^
Absolute structure: Flack (1983 ▶), 3453 Friedel pairs
Flack parameter: 0.031 (13)

### 

Data collection: *COLLECT* (Nonius, 1999 ▶); cell refinement: *SCALEPACK* (Otwinowski & Minor, 1997 ▶); data reduction: *DENZO* (Otwinowski & Minor, 1997 ▶) and *SCALEPACK*; program(s) used to solve structure: *SHELXS97* (Sheldrick 2008 ▶); program(s) used to refine structure: *SHELXL97* (Sheldrick, 2008 ▶); molecular graphics: *PLATON* (Spek, 2009 ▶); software used to prepare material for publication: *WinGX* (Farrugia, 2012 ▶).

## Supplementary Material

Crystal structure: contains datablock(s) I, global. DOI: 10.1107/S1600536812043590/su2514sup1.cif

Structure factors: contains datablock(s) I. DOI: 10.1107/S1600536812043590/su2514Isup2.hkl

Additional supplementary materials:  crystallographic information; 3D view; checkCIF report

## Figures and Tables

**Table 1:** Hydrogen-bond geometry (Å, °)

*D*—H⋯*A*	*D*—H	H⋯*A*	*D*⋯*A*	*D*—H⋯*A*
C5—H5⋯O22^i^	0.98	2.54	3.491 (6)	165
C18—H18*C*⋯O30^ii^	0.96	2.59	3.545 (7)	173
C27—H27*B*⋯Cl2^iii^	0.96	2.32	2.957 (18)	124
C32—H32*A*⋯O6^iv^	0.97	2.23	3.00 (2)	135
C32—H32*B*⋯Cl1^v^	0.97	2.16	2.89 (3)	130

Symmetry codes: (i) 
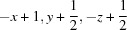
; (ii) 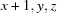; (iii) 
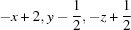
; (iv) 
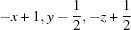
; (v) 
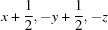
.

**Fig. 1.**
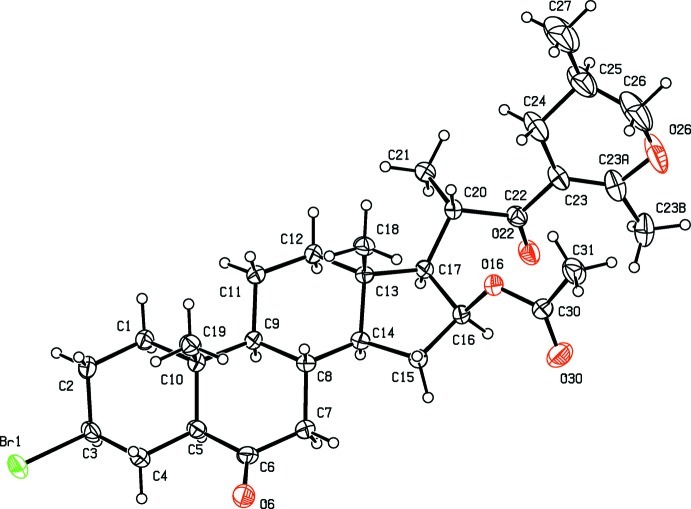
A view of the molecular structure of the title compound, with the atom numbering. Displacement ellipsoids are drawn at 30% probability level.

## supplementary crystallographic information

### Comment

Steroidal saponins are plant metabolites with a broad range of biological activities (Hostettmann & Marston, 1995). They are composed by a glycoside and a triterpene or steroidal fragment. Hydrolysis of saponins provides a glycoside free portion termed sapogenin which can be of the cholestane, furostane or spirostane type. The spirostane sapogenins also display economic importance due to their application in the synthesis of biologically active compounds such as insect hormones (Lee *et al.*, 1976) cephalostatins and ritterazines (Lee *et al.*, 2009, Phillips & Shair, 2007 and Pettit *et al.*, 1988). In previous studies we reported the preparation of epoxycholestane derivatives as useful intermediates in the synthesis of norbrassinosteroid analogues (Rincón *et al.*, 2006), in continuation with our studies we report herein on the synthesis and crystal structure of the title compound, (I), obtained by treatment of the previously reported (25*R*)-23-acetyl-3β-bromo-16β-acetoxy-22,26-epoxy-5α-cholest-22-en-6-one(Castro-Méndez *et al.*, 2002) with *p*-toluenesulfonic acid. In turn, the 22,26-epoxy-5α-cholestanic derivative was obtained in five steps using a modified procedure of the reported methodology (Castro-Méndez *et al.*, 2002).

The title compound is interesting because it is a useful intermediate to introduce functionality at the 2 and 3 positions of brassinosteroid analogues. The X-ray crystal structure analysis showed that the configuration at the stereogenic centers C20*S* and C25*R* are retained (Fig. 1). The steroid nucleus shows that the *A*/*B*, *B*/*C* and *C*/*D* rings junctions are *trans*. The presence of the bromine bonded to C3 does not disturb the chair conformation of the A ring [puckering parameters for ring (C1—C5/C10) are *Q* = 0.576 (5) Å, *θ* = 2.2 (5)°, *φ* = 323 (20)°; Cremer & Pople, 1975 ]. Ring *B* assumes an almost perfect chair conformation which contains a carbonyl group at C5 [puckering parameters: *Q* = 0.565 (5) Å, *θ* = 14.0 (5)°,*φ* = 278 (2)°, if the calculation starts from C5 to C10 and proceeds in counterclockwise direction]. The same chair conformation was observed for the *C* ring [puckering parameters (C8/C9/C11—C14) *Q* = 0.570 (5) Å, *θ* = 4.8 (5)°, *φ* = 251 (6)°]. The five-membered *D* ring has an envelope conformation with atom C13 as the flap [puckering parameters (C13/C14/C15/C16/C17) *q*_2_ = 0.480 (5) Å and *φ*_2_ = 188.7 (6)°]. The six-membered dihydropyran *E* ring adopts a twisted-boat conformation [puckering parameters (O26/C23A/C23—C26) *Q* = 0.472 (10) Å, *θ* = 122.8 (10)°, *φ* = 79.5 (11)°].

The bond distances for C6—O6 and C23—C23A are 1.205 (6) Å and 1.344 (9) Å, respectively, confirming the existence of a double bond. The C3—Br1 bond distance is 1.977 (5) Å being slightly longer than the average values reported for Br—C*= 1.966 (29) (Allen *et al.*, 1987) and C3—Br1 = 1.966 (5) Å in a related steroidal compound containing bromine in the same position (Castro-Méndez *et al.*, 2002). The bromine at position three is equatorial and antiperiplanar to the C4—C5 bond with a torsion angle -178.1 (3). The bond distances for C23A—O26 and C26—O26 are 1.365 (8) Å and 1.460 (13) Å, respectively (Table 1); these values are in the range reported for bond distances in a similar compound, that is the cholestane derivative from diosgenin [C22—O26, 1.365 (5) Å and C26—O26 1.441 (5) Å; Castro-Méndez *et al.*, 2002] and are in the average range reported for C*sp*^2^—O(2) in enol ethers C═ C—O—C*= 1.354 (16) Å and C*sp*^3^—O(2) in tetrahydropyran 1.441 (15) Å (Allen *et al.*, 1987).

In the crystal, molecules are linked by C—H···O and C—H···Cl hydrogen bonds (Table 1), the latter involve the dichloromethane solvent molecule, forming a three-dimensional supramolecular architecture.

### Experimental

Tosylation of diosgenin with TsCl in pyridine (Monroe *et al.*, 1956), followed by preparation of the i-steroid derivative using a methodology previously described (Steele *et al.*, 1963), oxidation with PDC (Corey & Suggs, 1975) and subsequent treatment with HBr/AcOEt (Iglesias-Arteaga *et al.*, 1998) gave 25*R*-3β-bromo-5α-spirostan-6-one which was transformed into (25*R*)-23-acetyl-3β-bromo-16β-acetoxy-22,26-epoxy-5α-cholest-22-en-6-one using ZnCl_2_ instead of the previously described methodology (Castro-Méndez *et al.*, 2002). Finally, the title compound was obtained by treatment of (25*R*)-23-acetyl-3β-bromo-16β-acetoxy-22,26-epoxy-5α-cholest-22-en-6-one (0.260 g, 0.493 mmol) with *p*-toluenesulfonic acid (0.260 g,1.36 mmol)in 0.7 ml toluene at 393 K for 30 minutes under vigorous stirring in a pressure tube. The solvent was evaporated under vacuum and the organic phase extracted with CH_2_Cl_2_-water, neutralized with NaHCO_3_ and dried over Na_2_SO_4_ to give a 0.160 g (61% yield) as white crystals which were purified by chromatography using a mixture of 70:30 hexane:ethyl acetate. (m.p. 468 – 470 K). Analysis calc.: C_31_H_45_O_5_Br: C 64.46, H 7.85, Br 13.85, O 13.85 %. Found: C 64.0, H 8.10 %. Block-like colourless crystals of the title compound, suitable for X-ray analysis, were grown by slow evaporation in a mixture of hexane:ethyl acetate (70:30) and a minimum quantity of CH_2_Cl_2_. Spectroscipic data for the title compound are given in the archived CIF.

### Refinement

All H atoms were placed in calculated positions and treated as riding atoms: C-H = 0.98, 0.97 and 0.96 Å for CH, CH_2_ and CH_3_ H atoms, respectively, with U_iso_(H) = k × Ueq(C), where k = 1.5 for CH_3_ H atoms and = 1.2 for other H atoms.

### Crystal data

**Table:**

C_31_H_45_BrO_5_·CH_2_Cl_2_	*D*_x_ = 1.406 Mg m^−^^3^
*M**_r_* = 662.51	Melting point: 468(2) K
Orthorhombic, *P*2_1_2_1_2_1_	Mo *K*α radiation, λ = 0.71073 Å
Hall symbol: P 2ac 2ab	Cell parameters from 600 reflections
*a* = 7.4423 (1) Å	θ = 3.5–28.7°
*b* = 15.6578 (2) Å	µ = 1.52 mm^−^^1^
*c* = 26.8496 (3) Å	*T* = 293 K
*V* = 3128.79 (7) Å^3^	Block, colourless
*Z* = 4	0.15 × 0.10 × 0.08 mm
*F*(000) = 1392	

### Data collection

**Table:**

Nonius KappaCCD diffractometer	5668 reflections with *I* > 2σ(*I*)
Radiation source: fine-focus sealed tube	*R*_int_ = 0.088
Graphite monochromator	θ_max_ = 28.7°, θ_min_ = 3.5°
Detector resolution: 9 pixels mm^-1^	*h* = −9→9
φ and ω scans	*k* = −20→21
37310 measured reflections	*l* = −36→36
7934 independent reflections	

### Refinement

**Table:**

Refinement on *F*^2^	Hydrogen site location: inferred from neighbouring sites
Least-squares matrix: full	H-atom parameters constrained
*R*[*F*^2^ > 2σ(*F*^2^)] = 0.068	*w* = 1/[σ^2^(*F*_o_^2^) + (0.087*P*)^2^ + 3.6522*P*] where *P* = (*F*_o_^2^ + 2*F*_c_^2^)/3
*wR*(*F*^2^) = 0.181	(Δ/σ)_max_ < 0.001
*S* = 1.03	Δρ_max_ = 0.75 e Å^−^^3^
7934 reflections	Δρ_min_ = −0.68 e Å^−^^3^
362 parameters	Extinction correction: *SHELXL97* (Sheldrick, 2008), Fc^*^=kFc[1+0.001xFc^2^λ^3^/sin(2θ)]^-1/4^
0 restraints	Extinction coefficient: 0.0038 (10)
Primary atom site location: structure-invariant direct methods	Absolute structure: Flack (1983), 3453 Friedel pairs
Secondary atom site location: difference Fourier map	Flack parameter: 0.031 (13)

### Special details

**Table:**

Experimental. Spectroscopic data for the title compound: UV λ_max_ 269 nm (ε 624); IR ν_max_ cm^-1^(KBr): 2959 (CH), 1737 (OAc), 1711 (C═O), 1665 (C═O), 1452 (CH_3_), 1334 (CH), 1247 (C—O), 988 (C═ C), 710 (CH_2_), MS, m/*z*: (%): 578 ([*M*^+^], 1.4), 206 (18), 205 (32), 191 (10), 166 (10), 140 (15), 139 (100), 43 (59); ^1^H NMR (300 MHz, CDCl_3_) δ: 5.02 (1*H*, m, H-16), 4.09 (1*H*, d, *J* = 11.0 Hz, H-26), 3.94 (1*H*, m, H-3), 3.40 (1*H*, t, *J* = 10.0 Hz, H-26), 3.20 (1*H*, dq, *J*_17–20_ = 10.6 Hz, *J*_20–21_ = 6.94 Hz, H-20), 2.06 (3*H*, s, 3-OCOCH_3_), 2.14 (3*H*, s, 23''-CH_3_), 1.90 (3*H*, s, 16-OCOCH_3_),1.10 (3*H*, d, *J* = 6.9 Hz, CH_3_-27), 1.02 (3*H*, d, *J* = 6.1 Hz, CH_3_-21), 0.88 (3*H*, s, CH_3_-19), 0.81 (3*H*, s, CH_3_-18). ^13^C NMR (100 MHz, CDCl_3_) δ: 203.9 (22-CO), 169.8 (16-OCOCH_3_), 164.9 (C-23'), 59.1 (C-5), 209.3(C-6), 107.6 (C-23), 75.4 (C-16), 50.6 (C-3), 71.9 (C-26), 56.1 (C-17), 54.2 (C-14), 53.8 (C-9), 42.9 (C-13), 39.2 (C-12), 38.7 (C-20), 31.8 (C-4), 33.5 (C-1), 40.8 (C-10), 34.4 (C-15), 46.4 (C-7), 37.3 (C-8), 30.9 (C-24), 32.5 (C-2), 26.9 (C-25), 21.3 (16-OCOCH_3_), 21.5 (C-11), 21.0 (C-23''), 19.6 (C-19), 17.3 (C-21), 17.1 (C-27), 13.3 (C-18).
Geometry. All s.u.'s (except the s.u. in the dihedral angle between two l.s. planes) are estimated using the full covariance matrix. The cell s.u.'s are taken into account individually in the estimation of s.u.'s in distances, angles and torsion angles; correlations between s.u.'s in cell parameters are only used when they are defined by crystal symmetry. An approximate (isotropic) treatment of cell s.u.'s is used for estimating s.u.'s involving l.s. planes.
Refinement. Refinement of *F*^2^ against ALL reflections. The weighted *R*-factor *wR* and goodness of fit *S* are based on *F*^2^, conventional *R*-factors *R* are based on *F*, with *F* set to zero for negative *F*^2^. The threshold expression of *F*^2^ > 2σ(*F*^2^) is used only for calculating *R*-factors(gt) *etc*. and is not relevant to the choice of reflections for refinement. *R*-factors based on *F*^2^ are statistically about twice as large as those based on *F*, and *R*-factors based on ALL data will be even larger.

### Fractional atomic coordinates and isotropic or equivalent isotropic displacement parameters (Å^2^)

**Table:**

	*x*	*y*	*z*	*U*_iso_*/*U*_eq_	
Br1	0.80129 (8)	0.99071 (3)	0.28226 (2)	0.0514 (2)	
O6	0.3019 (5)	0.7718 (2)	0.35688 (17)	0.0616 (13)	
O16	0.3802 (5)	0.2944 (2)	0.41187 (13)	0.0429 (11)	
O22	0.4244 (5)	0.1706 (2)	0.31163 (14)	0.0531 (11)	
O26	0.4238 (10)	−0.0251 (3)	0.4188 (2)	0.108 (3)	
O30	0.0825 (6)	0.2752 (3)	0.41123 (19)	0.0793 (18)	
C1	0.8950 (6)	0.7228 (3)	0.29478 (19)	0.0393 (14)	
C2	0.9275 (7)	0.8200 (3)	0.2955 (2)	0.0430 (14)	
C3	0.7592 (6)	0.8661 (3)	0.27962 (19)	0.0411 (13)	
C4	0.5983 (7)	0.8440 (3)	0.3109 (2)	0.0440 (16)	
C5	0.5683 (6)	0.7469 (3)	0.31053 (17)	0.0360 (12)	
C6	0.4036 (7)	0.7210 (3)	0.3384 (2)	0.0433 (14)	
C7	0.3660 (6)	0.6261 (3)	0.3402 (2)	0.0437 (14)	
C8	0.5314 (6)	0.5704 (3)	0.35181 (18)	0.0347 (12)	
C9	0.6928 (6)	0.5994 (3)	0.31974 (16)	0.0337 (11)	
C10	0.7360 (6)	0.6949 (3)	0.32813 (16)	0.0337 (12)	
C11	0.8546 (6)	0.5399 (3)	0.3259 (2)	0.0410 (14)	
C12	0.8068 (7)	0.4453 (3)	0.31787 (19)	0.0400 (14)	
C13	0.6532 (5)	0.4169 (3)	0.35206 (16)	0.0330 (12)	
C14	0.4942 (6)	0.4770 (3)	0.34192 (18)	0.0360 (12)	
C15	0.3379 (6)	0.4350 (3)	0.3697 (2)	0.0420 (14)	
C16	0.3743 (6)	0.3378 (3)	0.36390 (17)	0.0357 (12)	
C17	0.5626 (6)	0.3305 (3)	0.33936 (17)	0.0343 (12)	
C18	0.7143 (7)	0.4186 (3)	0.40677 (18)	0.0427 (14)	
C19	0.7786 (8)	0.7143 (3)	0.38279 (18)	0.0447 (16)	
C20	0.6614 (6)	0.2460 (3)	0.35157 (18)	0.0387 (14)	
C21	0.8164 (7)	0.2293 (3)	0.3151 (2)	0.0477 (16)	
C22	0.5302 (7)	0.1703 (3)	0.34636 (18)	0.0397 (14)	
C23	0.5464 (8)	0.1008 (3)	0.3826 (2)	0.0500 (18)	
C23A	0.4238 (10)	0.0378 (4)	0.3836 (2)	0.065 (2)	
C23B	0.2656 (11)	0.0256 (5)	0.3511 (3)	0.086 (3)	
C24	0.7091 (12)	0.0994 (3)	0.4160 (2)	0.071 (2)	
C25	0.7367 (14)	0.0126 (5)	0.4400 (3)	0.096 (3)	
C26	0.5590 (18)	−0.0187 (6)	0.4580 (3)	0.118 (5)	
C27	0.8847 (18)	0.0162 (7)	0.4812 (4)	0.136 (5)	
C30	0.2270 (7)	0.2641 (3)	0.4302 (2)	0.0470 (17)	
C31	0.2554 (9)	0.2139 (4)	0.4772 (2)	0.0613 (19)	
Cl1	0.6265 (19)	0.2328 (4)	0.0142 (2)	0.431 (7)	
Cl2	0.818 (2)	0.3949 (7)	0.0006 (3)	0.398 (8)	
C32	0.803 (4)	0.3144 (11)	0.0383 (7)	0.268 (15)	
H1A	1.00321	0.69403	0.30591	0.0472*	
H1B	0.87157	0.70498	0.26080	0.0472*	
H2A	0.96114	0.83786	0.32883	0.0512*	
H2B	1.02524	0.83427	0.27310	0.0512*	
H3	0.73308	0.85039	0.24504	0.0493*	
H4A	0.49272	0.87253	0.29774	0.0527*	
H4B	0.61698	0.86343	0.34475	0.0527*	
H5	0.54770	0.73093	0.27571	0.0434*	
H7A	0.31708	0.60855	0.30826	0.0525*	
H7B	0.27497	0.61540	0.36525	0.0525*	
H8	0.56241	0.57745	0.38705	0.0419*	
H9	0.65452	0.59414	0.28495	0.0403*	
H11A	0.90383	0.54698	0.35904	0.0490*	
H11B	0.94678	0.55619	0.30214	0.0490*	
H12A	0.77192	0.43659	0.28344	0.0479*	
H12B	0.91193	0.41045	0.32438	0.0479*	
H14	0.46691	0.47214	0.30632	0.0434*	
H15A	0.22363	0.45060	0.35490	0.0502*	
H15B	0.33747	0.45150	0.40454	0.0502*	
H16	0.28301	0.31166	0.34246	0.0427*	
H17	0.54264	0.33059	0.30329	0.0409*	
H18A	0.61701	0.40056	0.42778	0.0639*	
H18B	0.74936	0.47562	0.41564	0.0639*	
H18C	0.81448	0.38073	0.41106	0.0639*	
H19A	0.67965	0.69663	0.40329	0.0669*	
H19B	0.79785	0.77454	0.38681	0.0669*	
H19C	0.88495	0.68391	0.39254	0.0669*	
H20	0.70800	0.24819	0.38568	0.0459*	
H21A	0.90162	0.27518	0.31709	0.0720*	
H21B	0.77002	0.22565	0.28182	0.0720*	
H21C	0.87455	0.17653	0.32364	0.0720*	
H23A	0.30428	0.02034	0.31711	0.1293*	
H23B	0.18680	0.07382	0.35421	0.1293*	
H23C	0.20303	−0.02535	0.36083	0.1293*	
H24A	0.69499	0.14216	0.44181	0.0850*	
H24B	0.81472	0.11404	0.39661	0.0850*	
H25	0.77821	−0.02680	0.41402	0.1156*	
H26A	0.57485	−0.07443	0.47309	0.1415*	
H26B	0.51536	0.01984	0.48356	0.1415*	
H27A	0.99494	0.03671	0.46697	0.2046*	
H27B	0.90312	−0.03990	0.49463	0.2046*	
H27C	0.84698	0.05413	0.50726	0.2046*	
H31A	0.38116	0.21255	0.48510	0.0921*	
H31B	0.21244	0.15658	0.47259	0.0921*	
H31C	0.19064	0.24032	0.50403	0.0921*	
H32A	0.76877	0.33433	0.07112	0.3239*	
H32B	0.91948	0.28676	0.04100	0.3239*	

### Atomic displacement parameters (Å^2^)

**Table:**

	*U*^11^	*U*^22^	*U*^33^	*U*^12^	*U*^13^	*U*^23^
Br1	0.0619 (3)	0.0273 (2)	0.0649 (3)	−0.0021 (2)	−0.0020 (3)	0.0035 (2)
O6	0.046 (2)	0.0369 (18)	0.102 (3)	0.0036 (19)	0.017 (2)	−0.0094 (18)
O16	0.0381 (17)	0.0397 (18)	0.051 (2)	−0.0033 (14)	0.0011 (15)	0.0094 (15)
O22	0.066 (2)	0.0364 (18)	0.057 (2)	−0.0089 (18)	−0.018 (2)	0.0031 (16)
O26	0.160 (6)	0.051 (3)	0.114 (4)	−0.036 (3)	−0.010 (4)	0.034 (3)
O30	0.041 (2)	0.101 (4)	0.096 (3)	−0.006 (2)	−0.002 (2)	0.044 (3)
C1	0.033 (2)	0.032 (2)	0.053 (3)	−0.0008 (19)	0.007 (2)	−0.0002 (19)
C2	0.037 (2)	0.036 (2)	0.056 (3)	−0.008 (2)	0.003 (2)	−0.001 (2)
C3	0.050 (3)	0.0244 (18)	0.049 (2)	0.0002 (16)	−0.007 (2)	−0.0020 (19)
C4	0.044 (3)	0.031 (2)	0.057 (3)	0.002 (2)	−0.007 (2)	0.004 (2)
C5	0.038 (2)	0.029 (2)	0.041 (2)	0.0023 (19)	−0.007 (2)	−0.0004 (18)
C6	0.034 (2)	0.034 (2)	0.062 (3)	0.006 (2)	−0.006 (2)	−0.003 (2)
C7	0.031 (2)	0.036 (2)	0.064 (3)	0.0017 (19)	0.001 (2)	0.002 (2)
C8	0.030 (2)	0.034 (2)	0.040 (2)	0.0034 (18)	−0.0011 (19)	0.0040 (19)
C9	0.028 (2)	0.0321 (19)	0.041 (2)	−0.003 (2)	−0.004 (2)	0.0025 (16)
C10	0.029 (2)	0.032 (2)	0.040 (2)	−0.0030 (16)	−0.0012 (17)	0.0006 (17)
C11	0.029 (2)	0.033 (2)	0.061 (3)	0.0011 (17)	0.005 (2)	0.003 (2)
C12	0.029 (2)	0.032 (2)	0.059 (3)	−0.002 (2)	0.005 (2)	0.0023 (18)
C13	0.028 (2)	0.031 (2)	0.040 (2)	0.0033 (16)	0.0020 (18)	0.0032 (17)
C14	0.032 (2)	0.034 (2)	0.042 (2)	0.0021 (18)	−0.0021 (19)	0.0067 (19)
C15	0.033 (2)	0.035 (2)	0.058 (3)	0.0007 (19)	0.003 (2)	0.009 (2)
C16	0.032 (2)	0.032 (2)	0.043 (2)	−0.0039 (18)	−0.0040 (19)	0.0086 (18)
C17	0.032 (2)	0.030 (2)	0.041 (2)	−0.0015 (18)	−0.0055 (19)	0.0051 (18)
C18	0.040 (2)	0.038 (2)	0.050 (3)	0.002 (2)	−0.012 (2)	0.0044 (19)
C19	0.046 (3)	0.038 (2)	0.050 (3)	0.001 (2)	−0.005 (2)	−0.0005 (19)
C20	0.034 (2)	0.031 (2)	0.051 (3)	−0.0049 (18)	−0.003 (2)	0.0046 (18)
C21	0.041 (3)	0.033 (2)	0.069 (3)	0.004 (2)	0.002 (3)	0.001 (2)
C22	0.046 (3)	0.032 (2)	0.041 (2)	0.001 (2)	−0.003 (2)	−0.0031 (19)
C23	0.071 (4)	0.029 (2)	0.050 (3)	−0.001 (2)	−0.001 (3)	0.003 (2)
C23A	0.092 (5)	0.039 (3)	0.063 (4)	−0.015 (3)	0.006 (4)	0.000 (2)
C23B	0.083 (5)	0.057 (4)	0.119 (6)	−0.034 (3)	−0.003 (4)	0.007 (4)
C24	0.111 (5)	0.035 (3)	0.066 (3)	−0.009 (4)	−0.026 (4)	0.010 (2)
C25	0.135 (7)	0.056 (4)	0.098 (5)	−0.005 (5)	−0.048 (5)	0.025 (4)
C26	0.209 (12)	0.062 (5)	0.083 (5)	−0.015 (6)	−0.053 (7)	0.033 (4)
C27	0.188 (11)	0.085 (6)	0.136 (8)	−0.008 (7)	−0.084 (8)	0.048 (6)
C30	0.035 (3)	0.045 (3)	0.061 (3)	0.002 (2)	0.006 (2)	0.008 (2)
C31	0.072 (4)	0.054 (3)	0.058 (3)	0.000 (3)	0.012 (3)	0.021 (3)
Cl1	0.87 (2)	0.186 (5)	0.237 (6)	−0.123 (9)	0.347 (11)	−0.054 (4)
Cl2	0.547 (19)	0.378 (12)	0.269 (8)	0.110 (13)	0.089 (11)	0.049 (8)
C32	0.46 (4)	0.134 (13)	0.211 (16)	0.07 (2)	0.07 (2)	0.087 (13)

### Geometric parameters (Å, º)

**Table:**

Br1—C3	1.977 (5)	C2—H2B	0.9700
Cl1—C32	1.94 (3)	C2—H2A	0.9700
Cl2—C32	1.62 (2)	C3—H3	0.9800
O6—C6	1.205 (6)	C4—H4B	0.9700
O16—C30	1.329 (6)	C4—H4A	0.9700
O16—C16	1.457 (6)	C5—H5	0.9800
O22—C22	1.221 (6)	C7—H7B	0.9700
O26—C23A	1.365 (8)	C7—H7A	0.9700
O26—C26	1.460 (13)	C8—H8	0.9800
O30—C30	1.203 (7)	C9—H9	0.9800
C1—C10	1.547 (6)	C11—H11B	0.9700
C1—C2	1.541 (7)	C11—H11A	0.9700
C2—C3	1.507 (7)	C12—H12B	0.9700
C3—C4	1.503 (7)	C12—H12A	0.9700
C4—C5	1.537 (7)	C14—H14	0.9800
C5—C6	1.492 (7)	C15—H15B	0.9700
C5—C10	1.563 (6)	C15—H15A	0.9700
C6—C7	1.513 (7)	C16—H16	0.9800
C7—C8	1.541 (6)	C17—H17	0.9800
C8—C14	1.512 (7)	C18—H18B	0.9600
C8—C9	1.546 (6)	C18—H18A	0.9600
C9—C10	1.546 (7)	C18—H18C	0.9600
C9—C11	1.531 (6)	C19—H19B	0.9600
C10—C19	1.532 (7)	C19—H19C	0.9600
C11—C12	1.539 (7)	C19—H19A	0.9600
C12—C13	1.532 (7)	C20—H20	0.9800
C13—C18	1.538 (6)	C21—H21B	0.9600
C13—C14	1.536 (6)	C21—H21A	0.9600
C13—C17	1.550 (6)	C21—H21C	0.9600
C14—C15	1.530 (7)	C23B—H23C	0.9600
C15—C16	1.554 (7)	C23B—H23B	0.9600
C16—C17	1.553 (6)	C23B—H23A	0.9600
C17—C20	1.549 (7)	C24—H24A	0.9700
C20—C22	1.542 (7)	C24—H24B	0.9700
C20—C21	1.536 (7)	C25—H25	0.9800
C22—C23	1.465 (7)	C26—H26A	0.9700
C23—C24	1.507 (10)	C26—H26B	0.9700
C23—C23A	1.344 (9)	C27—H27B	0.9600
C23A—C23B	1.478 (11)	C27—H27C	0.9600
C24—C25	1.518 (9)	C27—H27A	0.9600
C25—C27	1.562 (15)	C31—H31C	0.9600
C25—C26	1.491 (16)	C31—H31A	0.9600
C30—C31	1.502 (8)	C31—H31B	0.9600
C1—H1A	0.9700	C32—H32A	0.9700
C1—H1B	0.9700	C32—H32B	0.9700
			
C16—O16—C30	117.9 (4)	H7A—C7—H7B	108.00
C23A—O26—C26	116.7 (6)	C7—C8—H8	109.00
C2—C1—C10	113.1 (4)	C9—C8—H8	109.00
C1—C2—C3	109.8 (4)	C14—C8—H8	109.00
Br1—C3—C2	109.3 (3)	C8—C9—H9	106.00
Br1—C3—C4	109.5 (3)	C10—C9—H9	106.00
C2—C3—C4	113.2 (4)	C11—C9—H9	106.00
C3—C4—C5	109.9 (4)	C9—C11—H11A	109.00
C4—C5—C6	112.6 (4)	C9—C11—H11B	109.00
C4—C5—C10	113.4 (4)	C12—C11—H11A	109.00
C6—C5—C10	111.3 (4)	C12—C11—H11B	109.00
O6—C6—C5	122.9 (4)	H11A—C11—H11B	108.00
O6—C6—C7	121.3 (5)	C11—C12—H12A	109.00
C5—C6—C7	115.8 (4)	C11—C12—H12B	109.00
C6—C7—C8	114.5 (4)	C13—C12—H12A	109.00
C7—C8—C9	110.0 (4)	C13—C12—H12B	109.00
C7—C8—C14	111.5 (4)	H12A—C12—H12B	108.00
C9—C8—C14	109.2 (4)	C8—C14—H14	107.00
C8—C9—C10	111.4 (4)	C13—C14—H14	107.00
C8—C9—C11	111.8 (4)	C15—C14—H14	107.00
C10—C9—C11	114.2 (4)	C14—C15—H15A	111.00
C1—C10—C5	106.8 (4)	C14—C15—H15B	111.00
C1—C10—C9	110.4 (4)	C16—C15—H15A	111.00
C1—C10—C19	109.9 (4)	C16—C15—H15B	111.00
C5—C10—C9	107.1 (4)	H15A—C15—H15B	109.00
C5—C10—C19	110.6 (4)	O16—C16—H16	110.00
C9—C10—C19	112.0 (4)	C15—C16—H16	110.00
C9—C11—C12	112.9 (4)	C17—C16—H16	110.00
C11—C12—C13	111.6 (4)	C13—C17—H17	106.00
C12—C13—C14	106.9 (4)	C16—C17—H17	106.00
C12—C13—C17	116.5 (4)	C20—C17—H17	106.00
C12—C13—C18	110.3 (4)	C13—C18—H18A	109.00
C14—C13—C17	99.2 (3)	C13—C18—H18B	109.00
C14—C13—C18	112.7 (4)	C13—C18—H18C	109.00
C17—C13—C18	110.7 (4)	H18A—C18—H18B	109.00
C8—C14—C13	114.9 (4)	H18A—C18—H18C	109.00
C8—C14—C15	118.0 (4)	H18B—C18—H18C	109.00
C13—C14—C15	103.6 (4)	C10—C19—H19A	109.00
C14—C15—C16	103.9 (4)	C10—C19—H19B	109.00
O16—C16—C15	111.9 (4)	C10—C19—H19C	109.00
O16—C16—C17	108.3 (4)	H19A—C19—H19B	109.00
C15—C16—C17	105.8 (4)	H19A—C19—H19C	109.00
C13—C17—C16	103.6 (4)	H19B—C19—H19C	109.00
C13—C17—C20	119.5 (4)	C17—C20—H20	110.00
C16—C17—C20	113.7 (4)	C21—C20—H20	110.00
C17—C20—C21	111.5 (4)	C22—C20—H20	110.00
C17—C20—C22	109.7 (4)	C20—C21—H21A	109.00
C21—C20—C22	106.7 (4)	C20—C21—H21B	109.00
O22—C22—C20	118.4 (4)	C20—C21—H21C	109.00
O22—C22—C23	124.3 (5)	H21A—C21—H21B	110.00
C20—C22—C23	117.3 (4)	H21A—C21—H21C	109.00
C22—C23—C23A	120.2 (5)	H21B—C21—H21C	109.00
C22—C23—C24	118.2 (5)	C23A—C23B—H23A	109.00
C23A—C23—C24	121.5 (5)	C23A—C23B—H23B	109.00
O26—C23A—C23	122.9 (6)	C23A—C23B—H23C	109.00
O26—C23A—C23B	108.4 (6)	H23A—C23B—H23B	109.00
C23—C23A—C23B	128.6 (6)	H23A—C23B—H23C	109.00
C23—C24—C25	112.0 (6)	H23B—C23B—H23C	109.00
C24—C25—C26	108.2 (8)	C23—C24—H24A	109.00
C24—C25—C27	111.3 (7)	C23—C24—H24B	109.00
C26—C25—C27	114.1 (8)	C25—C24—H24A	109.00
O26—C26—C25	113.6 (7)	C25—C24—H24B	109.00
O16—C30—O30	124.0 (5)	H24A—C24—H24B	108.00
O16—C30—C31	112.2 (5)	C24—C25—H25	108.00
O30—C30—C31	123.9 (5)	C26—C25—H25	108.00
C2—C1—H1A	109.00	C27—C25—H25	108.00
C2—C1—H1B	109.00	O26—C26—H26A	109.00
C10—C1—H1A	109.00	O26—C26—H26B	109.00
C10—C1—H1B	109.00	C25—C26—H26A	109.00
H1A—C1—H1B	108.00	C25—C26—H26B	109.00
C1—C2—H2A	110.00	H26A—C26—H26B	108.00
C1—C2—H2B	110.00	C25—C27—H27A	109.00
C3—C2—H2A	110.00	C25—C27—H27B	110.00
C3—C2—H2B	110.00	C25—C27—H27C	109.00
H2A—C2—H2B	108.00	H27A—C27—H27B	109.00
Br1—C3—H3	108.00	H27A—C27—H27C	109.00
C2—C3—H3	108.00	H27B—C27—H27C	110.00
C4—C3—H3	108.00	C30—C31—H31A	110.00
C3—C4—H4A	110.00	C30—C31—H31B	110.00
C3—C4—H4B	110.00	C30—C31—H31C	110.00
C5—C4—H4A	110.00	H31A—C31—H31B	109.00
C5—C4—H4B	110.00	H31A—C31—H31C	109.00
H4A—C4—H4B	108.00	H31B—C31—H31C	109.00
C4—C5—H5	106.00	Cl1—C32—Cl2	110.5 (13)
C6—C5—H5	106.00	Cl1—C32—H32A	110.00
C10—C5—H5	106.00	Cl1—C32—H32B	110.00
C6—C7—H7A	109.00	Cl2—C32—H32A	110.00
C6—C7—H7B	109.00	Cl2—C32—H32B	109.00
C8—C7—H7A	109.00	H32A—C32—H32B	108.00
C8—C7—H7B	109.00		
			
C30—O16—C16—C17	−154.6 (4)	C9—C11—C12—C13	−55.2 (5)
C30—O16—C16—C15	89.2 (5)	C11—C12—C13—C17	166.1 (4)
C16—O16—C30—O30	−5.1 (7)	C11—C12—C13—C18	−66.6 (5)
C16—O16—C30—C31	174.6 (4)	C11—C12—C13—C14	56.3 (5)
C23A—O26—C26—C25	−38.4 (10)	C18—C13—C14—C8	61.4 (5)
C26—O26—C23A—C23B	−173.4 (7)	C18—C13—C14—C15	−68.8 (5)
C26—O26—C23A—C23	3.5 (10)	C12—C13—C17—C16	−157.1 (4)
C2—C1—C10—C19	−64.4 (5)	C12—C13—C17—C20	75.2 (5)
C2—C1—C10—C9	171.6 (4)	C14—C13—C17—C16	−42.9 (4)
C10—C1—C2—C3	−57.2 (5)	C18—C13—C17—C16	75.8 (4)
C2—C1—C10—C5	55.6 (5)	C12—C13—C14—C15	169.9 (4)
C1—C2—C3—Br1	178.7 (3)	C18—C13—C17—C20	−51.9 (5)
C1—C2—C3—C4	56.4 (6)	C12—C13—C14—C8	−60.0 (5)
C2—C3—C4—C5	−55.9 (5)	C14—C13—C17—C20	−170.6 (4)
Br1—C3—C4—C5	−178.1 (3)	C17—C13—C14—C8	178.6 (4)
C3—C4—C5—C10	56.0 (5)	C17—C13—C14—C15	48.4 (4)
C3—C4—C5—C6	−176.5 (4)	C13—C14—C15—C16	−35.0 (5)
C4—C5—C6—C7	−179.4 (4)	C8—C14—C15—C16	−163.2 (4)
C6—C5—C10—C1	176.6 (4)	C14—C15—C16—O16	125.2 (4)
C4—C5—C6—O6	3.4 (7)	C14—C15—C16—C17	7.5 (5)
C10—C5—C6—O6	132.0 (5)	C15—C16—C17—C13	22.3 (4)
C6—C5—C10—C9	58.4 (5)	C15—C16—C17—C20	153.6 (4)
C6—C5—C10—C19	−63.9 (5)	O16—C16—C17—C20	33.4 (5)
C4—C5—C10—C19	64.3 (5)	O16—C16—C17—C13	−97.8 (4)
C4—C5—C10—C9	−173.4 (4)	C13—C17—C20—C21	−74.3 (5)
C10—C5—C6—C7	−50.7 (6)	C16—C17—C20—C21	162.8 (4)
C4—C5—C10—C1	−55.2 (5)	C13—C17—C20—C22	167.7 (4)
O6—C6—C7—C8	−137.9 (5)	C16—C17—C20—C22	44.8 (5)
C5—C6—C7—C8	44.8 (6)	C17—C20—C22—C23	−142.1 (4)
C6—C7—C8—C9	−46.2 (6)	C21—C20—C22—C23	97.0 (5)
C6—C7—C8—C14	−167.5 (4)	C21—C20—C22—O22	−80.6 (5)
C7—C8—C9—C11	−173.9 (4)	C17—C20—C22—O22	40.4 (6)
C14—C8—C9—C10	179.6 (4)	O22—C22—C23—C23A	−10.1 (8)
C7—C8—C14—C13	179.4 (4)	C20—C22—C23—C24	−11.3 (7)
C14—C8—C9—C11	−51.3 (5)	O22—C22—C23—C24	166.2 (5)
C7—C8—C14—C15	−57.9 (6)	C20—C22—C23—C23A	172.5 (5)
C7—C8—C9—C10	57.0 (5)	C24—C23—C23A—O26	9.0 (9)
C9—C8—C14—C13	57.7 (5)	C24—C23—C23A—C23B	−174.8 (6)
C9—C8—C14—C15	−179.6 (4)	C22—C23—C23A—C23B	1.3 (10)
C11—C9—C10—C1	53.5 (5)	C22—C23—C23A—O26	−174.9 (6)
C8—C9—C10—C19	58.5 (5)	C23A—C23—C24—C25	13.2 (9)
C11—C9—C10—C19	−69.3 (5)	C22—C23—C24—C25	−163.0 (6)
C11—C9—C10—C5	169.3 (4)	C23—C24—C25—C26	−44.2 (8)
C8—C9—C10—C5	−62.9 (4)	C23—C24—C25—C27	−170.3 (7)
C8—C9—C10—C1	−178.7 (4)	C24—C25—C26—O26	57.9 (9)
C10—C9—C11—C12	179.4 (4)	C27—C25—C26—O26	−177.7 (7)
C8—C9—C11—C12	51.8 (5)		

### Hydrogen-bond geometry (Å, º)

**Table:**

*D*—H···*A*	*D*—H	H···*A*	*D*···*A*	*D*—H···*A*
C5—H5···O22^i^	0.98	2.54	3.491 (6)	165
C18—H18*C*···O30^ii^	0.96	2.59	3.545 (7)	173
C27—H27*B*···Cl2^iii^	0.96	2.32	2.957 (18)	124
C32—H32*A*···O6^iv^	0.97	2.23	3.00 (2)	135
C32—H32*B*···Cl1^v^	0.97	2.16	2.89 (3)	130

Symmetry codes: (i) −*x*+1, *y*+1/2, −*z*+1/2; (ii) *x*+1, *y*, *z*; (iii) −*x*+2, *y*−1/2, −*z*+1/2; (iv) −*x*+1, *y*−1/2, −*z*+1/2; (v) *x*+1/2, −*y*+1/2, −*z*.
